# The latency time of SARS-CoV- 2 Delta variant in infection- and vaccine-naive individuals from Vietnam

**DOI:** 10.1186/s12879-025-10898-3

**Published:** 2025-04-12

**Authors:** Vera H. Arntzen, Manh Nguyen Duc, Marta Fiocco, Lan Truong Thi Thanh, Tam Nguyen Hoai Thao, Buu Mai Thanh, Tu-Anh Nguyen, Nhat Le Thanh Hoang, Marc Choisy, Lam Phung Khanh, Nga Le Hong, Ronald B. Geskus

**Affiliations:** 1 Mathematical Institute, Leiden University, Leiden, the Netherlands; 2 Centre for Tropical Medicine, Oxford University Clinical Research Unit, Ho Chi Minh City, Viet Nam; 3 Biomedical Data Science, Section of Medical Statistics, Leiden University Medical Center, Leiden, the Netherlands; 4 Statistics, Princess Maxima Center for Child Oncology, Utrecht, the Netherlands; 5 Department of Acute Infectious Disease Prevention and Control, Ho Chi Minh Center for Disease Control, Ho Chi Minh City, Viet Nam; 6 Centre for Tropical Medicine and Global Health, Nuffield Department of Clinical Medicine, University of Oxford, Oxford, UK

**Keywords:** SARS-CoV- 2, Latency time, Quarantine length, Doubly interval censored data, Truncation, Generalized gamma distribution

## Abstract

**Background:**

The latency time (from infection to infectiousness) guides the choice of measures required to control an infectious disease. Estimates of the SARS-CoV- 2 latency time are sparse due to lack of appropriate and representative data. Infection time is rarely known exactly and exposure information may be subject to several biases. Information on the endpoint requires repeated testing. Moreover, estimation is challenging because both the starting point and endpoint are typically interval censored and data may be subject to length-biased sampling (truncation).

**Methods:**

We collected detailed information on exposure from public health reports produced during an outbreak with the SARS-CoV- 2 Delta variant in Ho Chi Minh City, Vietnam, in May-July 2021. Using a custom digital form and application facilitated reliable choices on exposure window. This comprehensive data set on exposure and test results from 1951 individuals, collected in the absence of large-scale vaccination or earlier infection, is the first of its kind outside of China.

We accounted for the doubly interval censored nature of the observations and went beyond the standard assumption of a constant infection risk over calendar time (exponential growth) and allowed for flexibility regarding the latency time (generalized gamma distribution). We addressed right truncation due to a cutoff in data collection and a finite quarantine length. Employing a Bayesian approach, using the program JAGS, made the analyses relatively straightforward.

**Results:**

Assuming exponential growth, our estimate of SARS-CoV- 2 Delta variant’s mean latency time was 3.22 (95% Credible Interval 2.89 - 3.55) days; the median was 1.81 (95% CrI 1.44- 2.16) days; the 95 th percentile was 10.98 (95% CrI 9.91 - 12.41) days. These values were much larger if a uniform infection risk was assumed.

**Conclusions:**

Using a Bayesian approach with the JAGS program, we were able to estimate the SARS-CoV- 2 latency time distribution of the Delta variant in infection-naive and vaccine-naive individuals. Estimates were sensitive to the assumptions made regarding the risk of infection within the exposure window. Compared to earlier studies, the median latency time was shorter, while the 95 th percentile was larger. Our results stress the importance of thoughtful data collection and analysis for evidence-based control of an infectious disease.

**Supplementary Information:**

The online version contains supplementary material available at 10.1186/s12879-025-10898-3.

## Background

Understanding the natural history of SARS-CoV- 2 has been essential to shape public health measures. Quarantine length is ideally informed by the distribution of latency time (time from infection to onset of infectiousness). Making well-informed decisions with respect to quarantine length is important, because quarantining is burdensome for individuals. Furthermore, it is demanding in terms of logistics, especially when carried out in a designated facility, as was the policy in countries like Vietnam. More generally, the latency time distribution, together with the basic reproduction number $R_0$ and the extent of asymptomatic transmission, determine the efforts required to control spread of an infectious disease [1].

For SARS-CoV- 2, the quarantine length has been based on estimates of the incubation time distribution (time from infection to symptom onset). The reason is that only few studies estimated the SARS-CoV- 2 latency time distribution. These estimates were based on data from China and concerned the Delta variant (*N*= 93 [2], *N*= 40 [3] and *N*= 672 [4]), the Omicron variant (*N*= 467 [5] and *N*= 885 [4]) or unknown variant(s) (*N*= 177 [6]). In two of these studies, the majority of the included individuals was vaccinated (70% [3], 57% for Delta and 92% for Omicron infected individuals [4]), while in one study 27.6% of the individuals had received at least one vaccine dose [2]. For the other two studies vaccination status was unclear [5, 6].

Data needed to estimate the SARS-CoV- 2 latency time is difficult to collect. In order to get reasonably accurate information on the time of infection, possible exposure events need to be rare and number of infections needs to be low. Ideally all infected individuals in a population are traced. Exposure information is collected retrospectively by interviewing notified cases as part of a contact tracing policy. For start of infectiousness, start of RNA shedding is typically used as proxy [2, 4, 6]. We do the same, and call it start-of-shedding in this paper. Latency time can only be estimated if PCR- or antigen tests are performed repeatedly during follow-up. When an individual first tests positive by a PCR- or rapid antigen test, we assume that shedding has started. Such data was collected in Vietnam, where the government implemented a policy of quarantining in designated facilities. Since viral shedding patterns differ per SARS-CoV- 2 variant [7], the same is likely to hold for the latency time distribution.

Observations of latency time are typically doubly interval censored, which means that both the starting point and endpoint are at best known to fall in a time window. This complicates the analysis. To simplify estimation, common practice is to assume that the risk of infection within the exposure window is constant and that the latency time follows a gamma, lognormal or Weibull distribution. However, coronaviruses tend to have a long tailed incubation time distribution [8], and the same is likely to hold for the latency time distribution. Misspecification of the type of distribution can introduce bias in the estimates [9]. This is particularly problematic as the tail of the distribution informs quarantine length.

In this paper, we report our estimate of the latency time distribution of the SARS-CoV- 2 Delta variant in mostly infection-naive and vaccine-naive individuals using a unique data set from Vietnam. It covers the period from April 29 th until July 15 th, 2021, which defines the start of the first major outbreak in Ho Chi Minh City. We give a detailed description of the data collection process. We relax assumptions made in earlier studies by using observed trends in first positive test results during the outbreak in Ho Chi Minh City as basis for the infection risk distribution and by choosing the more flexible generalized gamma distribution for the latency time. We also address sampling bias due to right truncation, which causes longer latency times to be underrepresented in our sample. We describe a surprisingly simple implementation of the estimation procedure.

## Methods

### Data collection

Vietnam initially strived to prevent any SARS-CoV- 2 outbreak [10–12]. The country’s public health response included almost complete border closure, extensive contact tracing using an epidemiological classification system known as the ‘F-system’ [13] and enforced quarantining, as described in Appendix 1. As a consequence, Ho Chi Minh City barely reported any community transmission until May 2021.

Transmission considerably increased from mid-May 2021 onwards. Infections were predominantly, and from June onwards solely, from the AY.57 lineage of the Delta variant, probably originating from a single introduction in the second half of April 2021 [14]. Individuals who were infected with SARS-CoV- 2 in our analysis data set were mostly naive, i.e. they did not contract SARS-CoV- 2 before and had not built up immunity from vaccination.

**Fig. 1:**
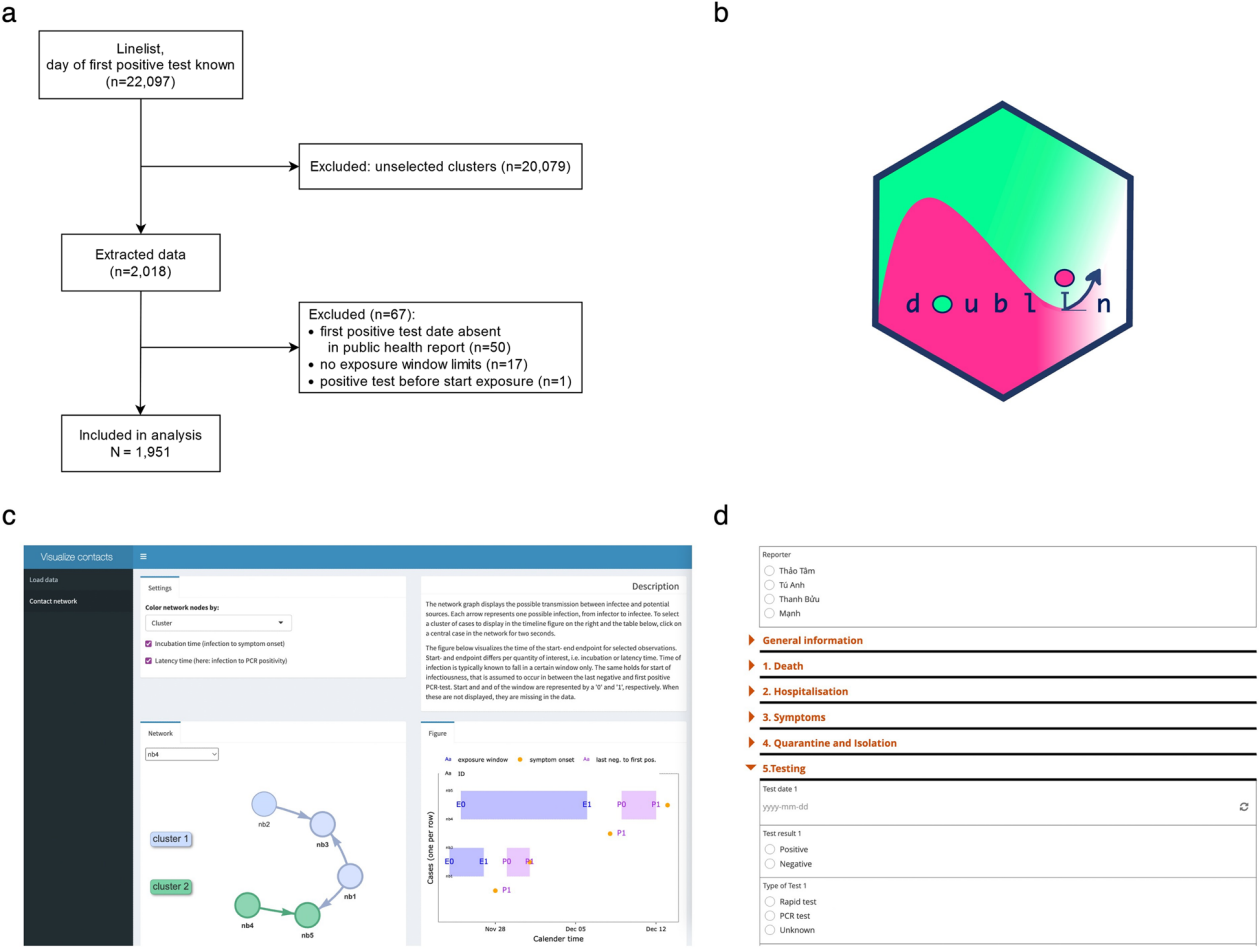
Illustration of the data collection process. **a** data flow from line list to data. After selection of clusters and exclusion of individuals without information on exposure or first test date in public health reports, 1,951 individuals remained for analysis. **b** the icon of the R package ‘doublIn’. **c** some of the R Shiny app functionalities (example data). **d** an excerpt from the KoboToolbox form used for data entry

Data originated from several documents, provided by the Ho Chi Minh City Center for Disease Control (HCDC). Figure 1a gives an overview. The starting point was a line list containing basic information on 22,097 individuals who tested positive between April 27 th and July 15 th, 2021. It covers nearly all of the infections in Ho Chi Minh City in that period. We took reporting delay into account, on average three days between testing and reporting, by excluding all first positive test results after July 12 th. Additional case information about exposure to potential sources was obtained from individual questionnaires taken upon confirmation of infection. This information was collected in 2827 public health reports as part of the contact tracing policy. Each report covered one or multiple PCR-confirmed cases. We received the files in different folders, with each folder mostly but not exclusively covering a specific cluster of transmission. For the remainder of this paper we refer to ‘cluster’ as cases that were grouped in the same folder.

Three persons from HCDC and one data coordinator from the Oxford University Clinical Research Unit (OUCRU) extracted the relevant case information from the reports. They used a list with all clusters to divide their work. If someone found a link between the respective cluster and another cluster, they would work on the related cluster next. For training purposes, all four persons first worked on the same cluster of roughly 80 individuals. They discussed their findings and standardized their data retrieval methods. Afterwards, they worked independently, while the coordinator from OUCRU performed random cross-checks of the data retrieved by the others.

To support the data extraction from the public health reports and reduce the risk of data entry errors, a KoboToolbox form with drop-down menus was developed [15] (Fig. 1d; full form available via https://ee.kobotoolbox.org/x/dcXRd59G). The relevant information from a report was entered into the KoboToolbox form for individuals who met the following criteria: at least one potential source was mentioned in the report or it provided at least one of either the test date (PCR- or antigen test) or the date of symptom onset. While working on a cluster, potential transmission trees were drawn on paper to assist with determining the exposure windows.

In some cases there were multiple options with respect to the exposure window. For instance, consider two cases A and B. They were exposed to a possible source between $t_1$ and $t_2$ and separated from each other at a later time $t_3$. Assuming that A and B contracted the infection from the same source led to the narrower window of $t_1$ to $t_2$ (called the strict choice in the sequel). Considering the possibility that A was initially infected from the source and subsequently transmitted the infection to B or vice versa led to the broader window of $t_1$ to $t_3$ (called the loose choice in the sequel).

We utilized the KoboconnectR R package to directly import data from KoboToolbox into R. To check and support the choice of the exposure windows, we developed an R Shiny application that visualizes the contact network based on entered data and the individual exposure information (see Fig. 1c for a glimpse of this app). The software for both the Shiny app and the KoboToolbox form can be found at https://github.com/manhnguy/Contact-Tracing-for-Respiratory-Transmitted-Diseases. The Shiny app can be conveniently run using the R package doublIn (Fig. 1b).

Halfway July 2021, as the city experienced a dramatic increase in the number of new cases, the contact tracing system became overwhelmed and not all cases could be described timely in public health reports. We included 75 of more than 100 clusters described by the public health reports (*N*= 2018 individuals). For 1968 of these individuals a first positive test result was present in the public health reports. It was mostly of unknown type, i.e. either the PCR or the rapid antigen test (2797 out of 2950). For all individuals the SARS-CoV- 2 infection was confirmed by a PCR test at some point during follow-up. For 1952 individuals we had at least one date of positive test as well as at least one of the exposure window limits. Excluding one case for whom the first positive test occurred before any exposure window (loose or strict choice) started, we ended up with 1951 for analysis (Fig. 1a).

### Statistical analyses

The data representation of an individual’s latency time observation consists of an exposure window and a start-of-shedding window. Let $E_{l}$ and $E_{r}$ be the boundaries of the exposure window in calendar time; likewise, let $S_{l}$ and $S_{r}$ denote the start-of-shedding window. For each individual, we prepared the data for analysis in six subsequent steps:

1. if the end of exposure ($E_r$) is missing, set it to the day of the first positive test;
2. if the start of exposure ($E_l$) is missing, set it to April 30, 2021, which is seen as the start of the Delta outbreak in Vietnam;
3. if the last negative test date ($S_l$) is missing, set it to the start of the exposure window ($S_l \leftarrow E_l$);
4. guarantee doubly interval-censored observations by considering a window width of at least one day in case date of infection or start-of-shedding are known exactly ($E_l - 0.5; E_r + 0.5; S_l - 0.5; S_r + 0.5$);
5. if the exposure window ends after the first positive test ($E_r> S_r$), set the end of exposure equal to the first positive test date ($E_r \leftarrow S_r$);
6. if the last negative test occurs before the start of exposure ($S_l < E_l$), set the last negative test equal to the start of exposure ($S_l \leftarrow E_l$).

**Fig. 2:**
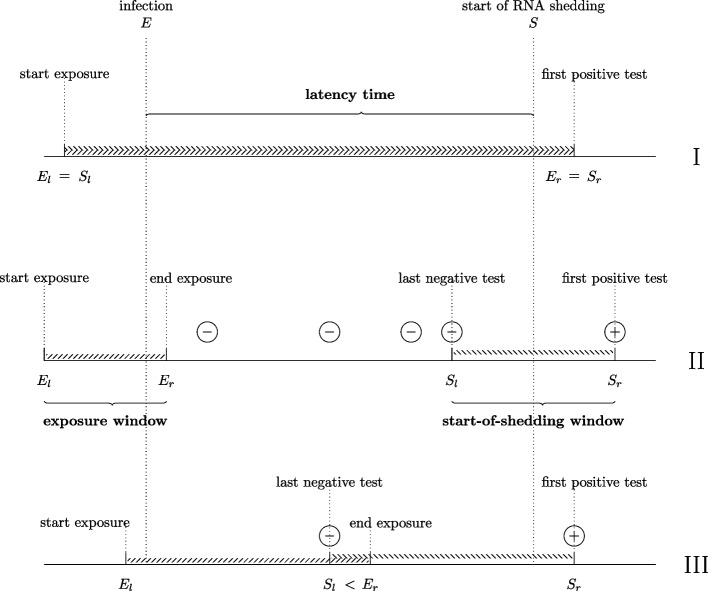
Illustration of observations of the latency time. Data representation for three individuals, each with an equal latency time $T=S-E$, observed differently with respect to double interval censoring. Time of infection is contained in the exposure window, which runs from the earliest possible moment of infection ($E_l$) to the latest ($E_r$). RNA shedding occurs between the last negative test result and the first positive test result, where ‘positive’ refers to the detected presence of SARS-CoV- 2 RNA (or antigen). The exposure window and start-of-shedding window may completely coincide ($E_l = S_I$ and $E_r = S_r$, indicated by I), not overlap ($E_r < S_l$, indicated by II) or partially overlap ($E_r> S_l$, indicated by III)

Building on previous work [16, 17], the exposure and start-of-shedding windows may completely coincide, not overlap, or partially overlap (Fig. 2).

We assume that the distributions of calendar time of infection (*E*) and latency time (*T*) are independent. Let $g(e|E_l=e_l,E_r=e_r)$ denote the density function of infection given the exposure window, which we shorten to $g(e|e_l,e_r)$. Let *f*(*t*) and *F*(*t*) denote the density and cumulative incidence of the latency time distribution respectively. We first consider the setting without truncation. The likelihood contribution for an observation *i* with complete overlap of both windows (Fig. 2, type I) is

1 $$\begin{aligned} l_i^{I} = \int _{e = e_{il}}^{e_{ir}} \int _{s = e}^{e_{ir}} g(e|e_{il},e_{ir}) f(s-e)dsde. \end{aligned}$$

When there is no overlap between the exposure window and the start-of-shedding window (Fig. 2, type II), the contribution to the likelihood is

2 $$\begin{aligned} l_i^{II} = \int _{e=e_{il}}^{e_{ir}} \int _{s=s_{il}}^{s_{ir}} g(e|e_{il},e_{ir}) f(s-e) dsde \end{aligned}$$

When both windows partially overlap (Fig. 2, type III) the likelihood is decomposed in four parts using (1) and (2) (see Appendix 2).

For the risk of infection within the exposure window $g(e|e_{il}, e_{ir})$ we assumed each individual to follow the same type of distribution. We considered the commonly used uniform distribution $g(e|e_{il}, e_{ir}) \sim \textrm{Unif}(e_{il}, e_{ir})$, assuming that the infection risk is constant over time. However, the start of an outbreak is characterised by exponential growth and violation of this assumption may lead to biased estimates [9]. Our alternative assumption is an exponential growth for the infection risk at the population level, which is passed on to the individual level. The probability of infection *I* is assumed to grow as follows: $I(t) = I(t_0) e^{r(t-t_0)}$, where $I(t_0)$ is the probability when one started counting cases from the outbreak ($t = t_0$) and *r* is the epidemic growth rate (per day). The epidemic growth rate *r* and the standard error (*SE*) were obtained by using the R package incidence [18]. In the estimation of the latency time we allow for uncertainty by assuming a normally distributed exponential growth rate with mean *r* and variance $SE^2$. Following Xin et al. [6], we performed sensitivity analyses where we consider a less and more extreme scenario by extracting and adding $\approx 2 SE$ to the estimated mean *r*, respectively.

For the distribution of latency time, we assumed the three-parameter generalized gamma distribution. We used the parameterisation as proposed by Stacy and Mihram [19] (Appendix 3). We also considered gamma and Weibull distributions as special cases.

**Fig. 3:**
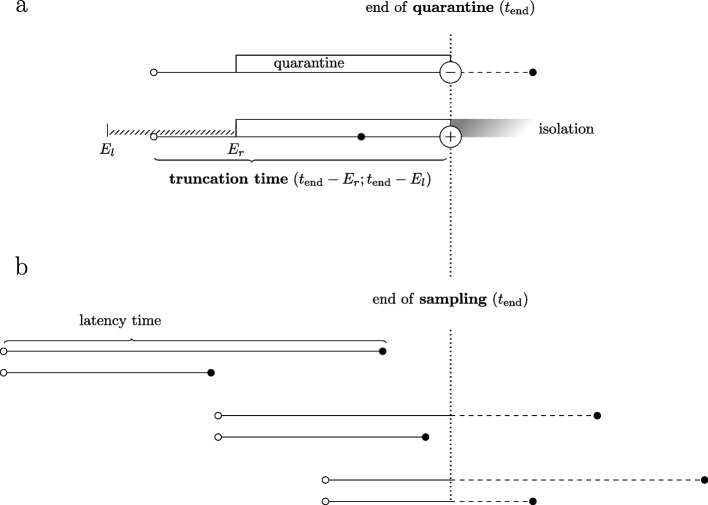
Illustration of truncation in the context of latency time estimation. Five different situations comparing two individuals, one with a long latency time (upper individual) and one with a short latency time (lower individual). **a** both individuals were infected (open bullet) on the same calendar day and entered quarantine on the same day. However, the upper individual is unobserved (left quarantine while still testing negative) whereas the lower individual tested positive by the end of quarantine and therefore appears in the data set. Because infection is not observed exactly but is known to fall within the exposure window ($E_l; E_r$), the truncation time is interval censored. **b** the same pair of latency times as in (**a**), but with three different choices of the calendar time of infection. The mechanism leading to truncation is the same as in (**a**). Individuals that end with a dashed line are not observed because the event occurs after the end of sampling

Our aim was to estimate the intrinsic latency time distribution [20], which is representative for all individuals under stable conditions. However, the likelihood terms (1) and (2) do not represent the intrinsic distribution due to the presence of right truncation in our data. The longer the time between infection and first positive test, the less likely it is that the individual is included. Individuals can be missed for two different reasons: (i) the repeated testing for SARS-CoV- 2 infection ended upon discharge from quarantine (Fig. 3a); (ii) cases that first tested positive after July 12 th were not included in our analysis (Fig. 3b). The latency time distribution is biased downwards if this phenomenon is not properly addressed [21, 22].

For the truncation date $t_{i, \textrm{end}}$ we used the start of quarantine plus the quarantine length of 21 days or July 12 th, whichever occurred earlier. The start of quarantine was known for 1038 individuals (53%); July 12 th was chosen for the others. An individual *i* was included in our data because the time of first positive test was earlier ($s_{ir} \le t_{i, \textrm{end}}$). The probability for this to happen is

3 $$\begin{aligned} \int _{e_{il}}^{e_{ir}}g(e|e_{il}, e_{ir})F(t_{i, \textrm{end}} - e)de \, , \end{aligned}$$

which is added to the likelihood as a denominator term.

For estimation a Bayesian approach was employed, using Markov Chain Monte Carlo methods to quantify the posterior distribution. Inspired by the code to estimate the incubation time for mpox [23], we wrote a program to allow for doubly interval censored data, assuming the distribution of infection within the exposure window to be known. We assumed non-negative, flat priors for all parameters (upper half of normal distribution with $\mu$ = 0 and $\sigma ^2 = 1000$) and used JAGS to quantify the posterior distributions [24]. Three chains were generated, each with 500000 iterations, a burn-in period of 10000 and a thinning factor of 10. The JAGS code that implements our doubly interval censored data setting with right truncation is surprisingly concise; there is no need to write down the complete likelihood (see Appendix 4 for details). We performed a simulation study to check the correctness of our implementation of right truncation (Fig. 8 in the Appendix 4). We also validated our code against the coarseDataTools package using simulated data, employing Weibull and gamma for the time-to-event distribution, uniform distribution for infection times, and no right truncation. Estimates were similar (results not shown).

All analyses were performed within RStudio [25], using the rjags interface to JAGS. We used the ALICE computing resources provided by Leiden University. We report estimates of mean, median, and the 95 th percentile, along with the corresponding 95% credible intervals. Parameter estimates that characterize the distributions can be found in the Supplementary Table.

Sensitivity analyses were performed by considering two scenarios i) only observations with narrow ($\le 4$ days) exposure windows were included and ii) truncation was not addressed.

## Results

**Fig. 4:**
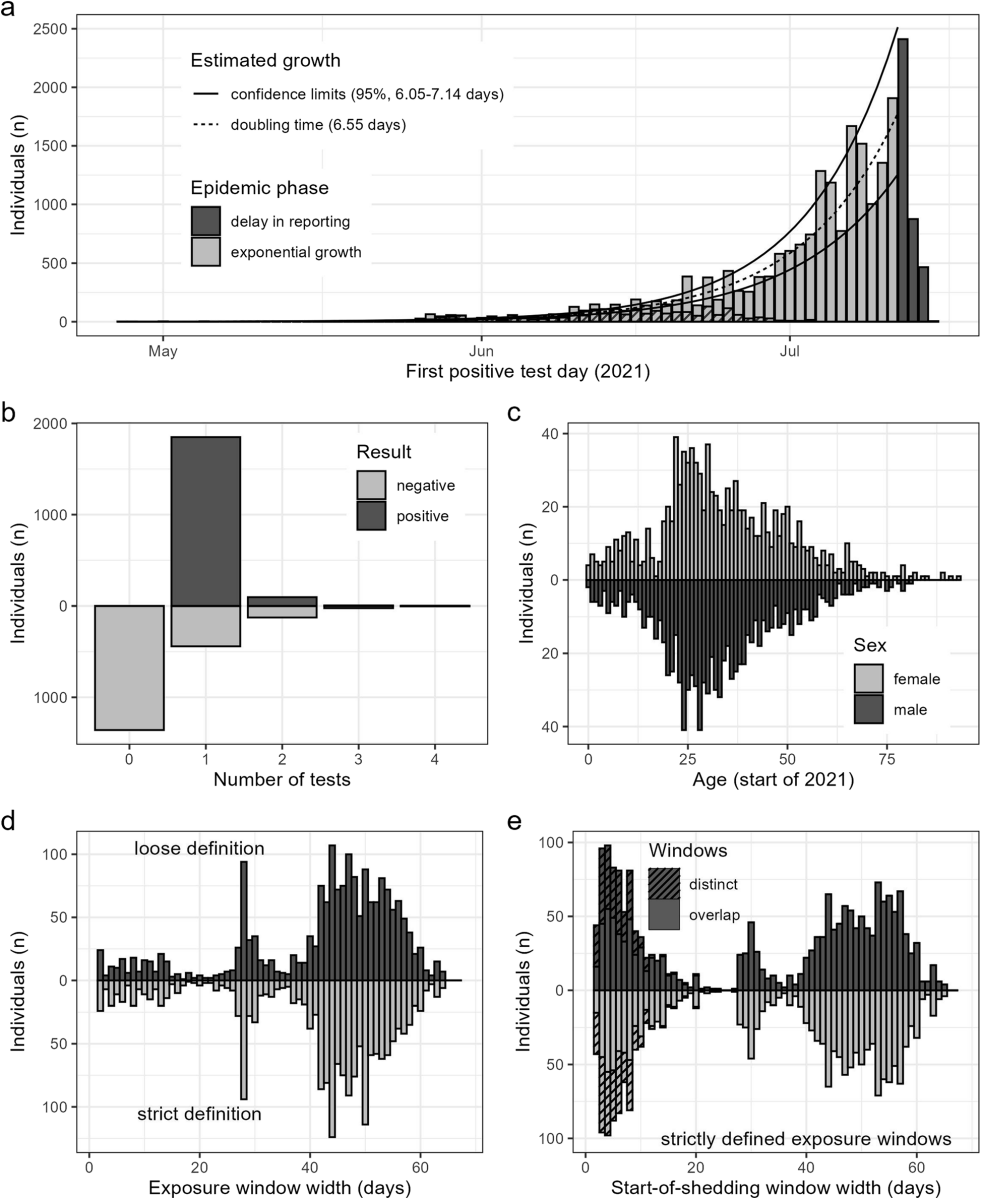
Data characteristics. **a** number of individuals from the line list that tested positive for SARS-CoV- 2 for the first time (per day on which the sample was taken). The dashed line represents the estimate of the exponential growth curve, with 95% confidence bounds (solid lines). Days after July 12 were excluded from the estimate because of reporting delay. The dashed area of each bar represents individuals included in our estimate of the latency time distribution. **b** distribution of the total number of positive and negative tests per individual used in the analysis (*N*= 1951). **c** age distribution by sex of individuals included in the analysis. **d** distribution of the width of exposure windows of individuals included in the analysis, based on the strict choice (below zero) and the loose choice (above zero). **e** distribution of start-of-shedding windows of individuals included in the analysis, using different choices of the exposure window (see above). Dashed areas indicate observations for whom exposure and start-of-shedding windows do not overlap

Figure 4a visualizes the calendar day of first positive test of all 22,097 individuals in the line list. Excluding 3759 individuals with a first positive test after July 12 th (dark grey bars), the estimate of the exponential growth factor *r* was 0.106 (95% CI 0.097; 0.114) and of the doubling time it was 6.55 days (95% CI 6.05; 7.14).

About one third of the 1951 individuals included in our analysis (*n*= 592; 30%) had at least one negative test result (Fig. 4b). The vast majority had a single positive test result (*n*= 1850; 95%), and had a first positive test before July 2021 (*N*= 1919; 98%; dashed bars in Fig. 4a). Males and females had a similar age distribution (Fig. 4c). Around 12% (*N*= 235) of individuals had an exposure window smaller than ten days using the strict choice, which decreased to 9.8% (*N*= 192) with the loose choice (Fig. 4d). The wider the start-of-shedding window, the more likely it overlapped with the exposure window (Fig. 4e). The start-of-shedding windows tended to be narrower than the exposure windows.

**Fig. 5:**
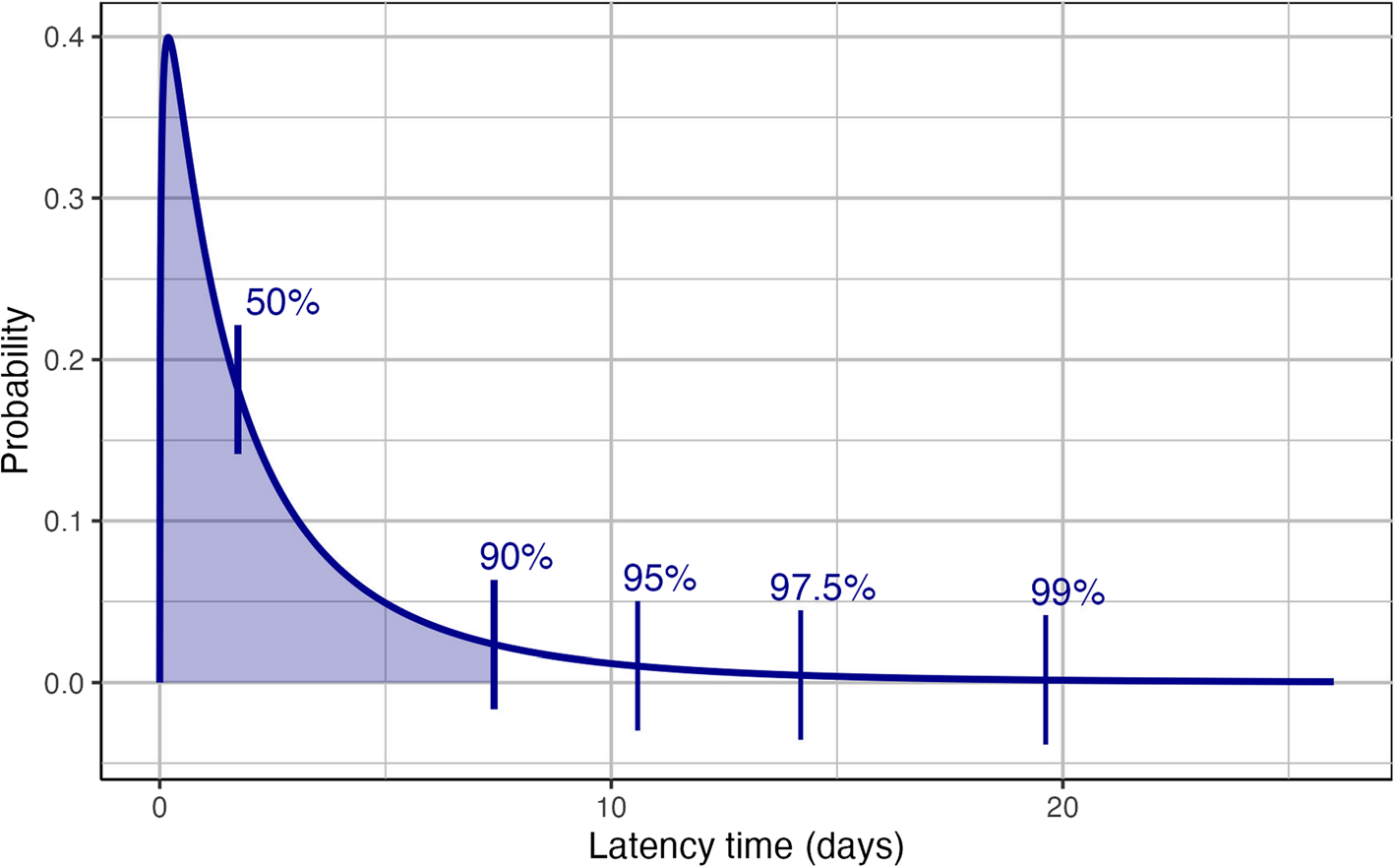
Estimated latency time distribution for SARS-CoV- 2, assuming a generalized gamma distribution), an exponential growth rate of r= 0.106 and using the strict exposure window bounds. Vertical bars correspond to the 50 th, 90 th, 95 th, 97.5 th and 99 th percentile

**Fig. 6:**
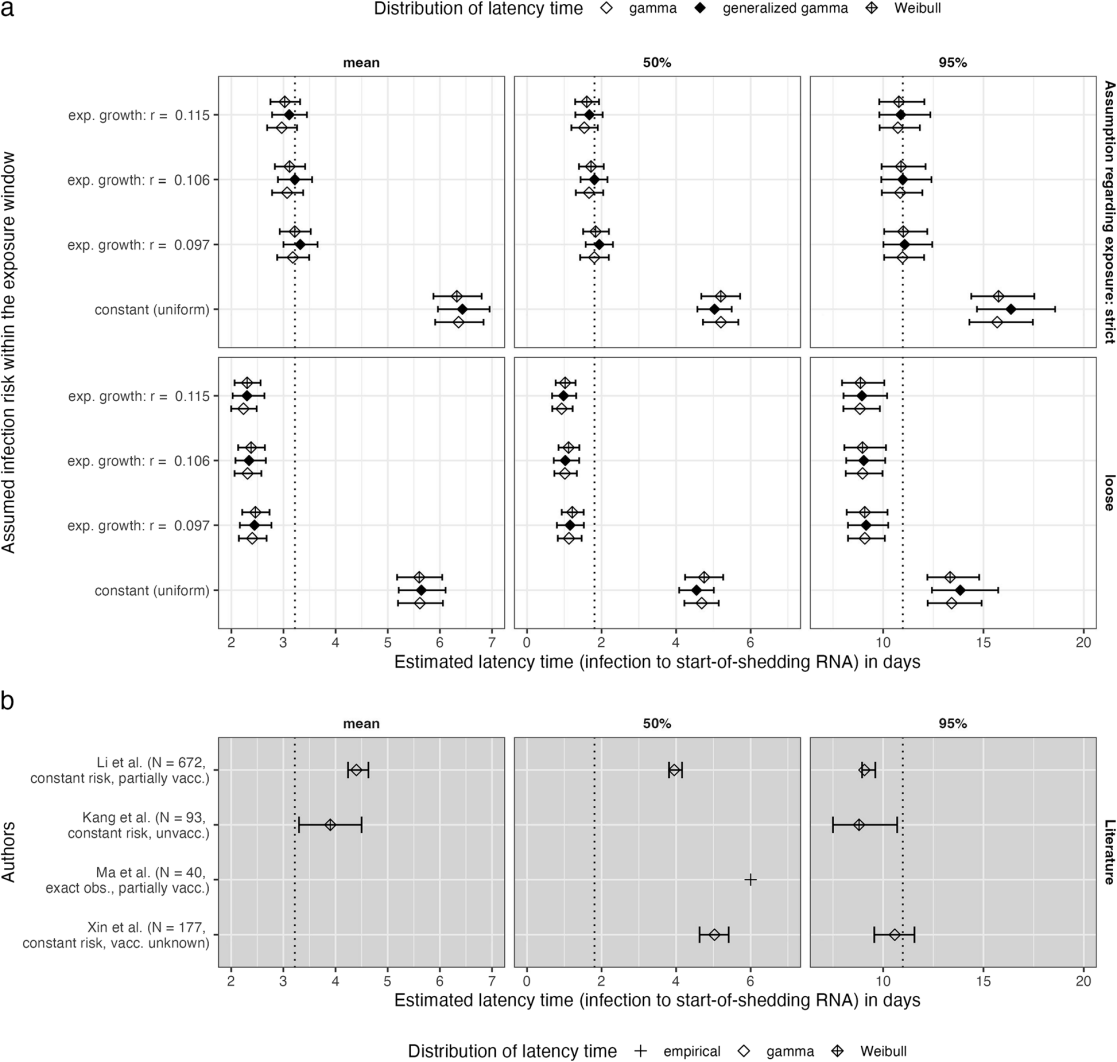
Latency time estimates for SARS-CoV- 2. **a** estimates based on our data concerning mostly the Delta variant; **b** estimates from earlier studies concerning the Delta variant (Xin: unknown variant). The mean, median and 95 th percentile are shown, using error bars to represent corresponding 95% credible intervals (**a**) or credible/confidence intervals (**b**). Symbols refer to the assumed parametric shape of the distribution of latency time. All estimates are given in days (x-axis). In (**a**), estimates are given for different assumptions for the infection risk within the exposure window (y-axis within panel) and exposure window choices (rows). Vertical dotted line gives our recommended estimate based on the generalized gamma distribution, an exponential increase in infection risk with growth factor r= 0.106 and a strict definition of the exposure window

**Fig. 8:**
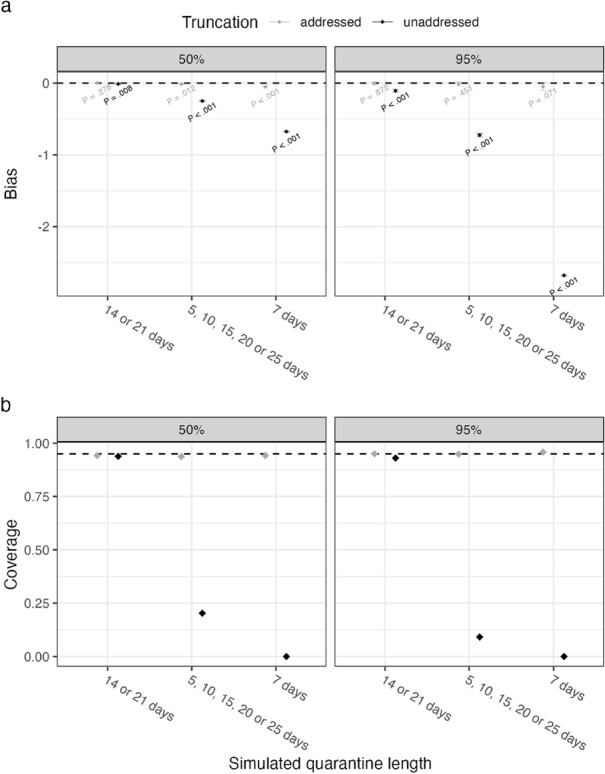
Simulation results to test our JAGS implementation. Estimates of median and 95 th percentile are shown. Grey and black color represent whether or not truncation was addressed in the analysis. Figure (**a**) visualizes the estimated bias, its 95% confidence interval and the p-value for testing the null hypothesis that the bias is zero. Figure (**b**) presents the coverage probability of the 95% confidence intervals, i.e. the proportion of data sets for which the confidence interval covered the true value

Assuming an exponential growth rate of r= 0.106 and using the strict choice of exposure window, we estimated the latency time distribution to have mean 3.22 (95% CrI 2.89; 3.55) days, median 1.81 (95% CrI 1.44; 2.16) days and 95 th percentile 10.98 (95% CrI 9.91; 12.41) days (Figs. 5 and 6a, dotted line). Estimates were similar if the weaker and stronger exponential growth scenarios were used (r $\pm \approx$2SE, i.e. r= 0.097 and r= 0.115). All estimates were considerably larger when a constant infection risk was assumed. The differences between generalized gamma, gamma and Weibull distributions were negligible. The estimates with a loose choice of the exposure window were slightly smaller (Fig. 6a, second row). Figure 6b reports estimates from earlier studies on the Delta or unknown variant.

**Fig. 9:**
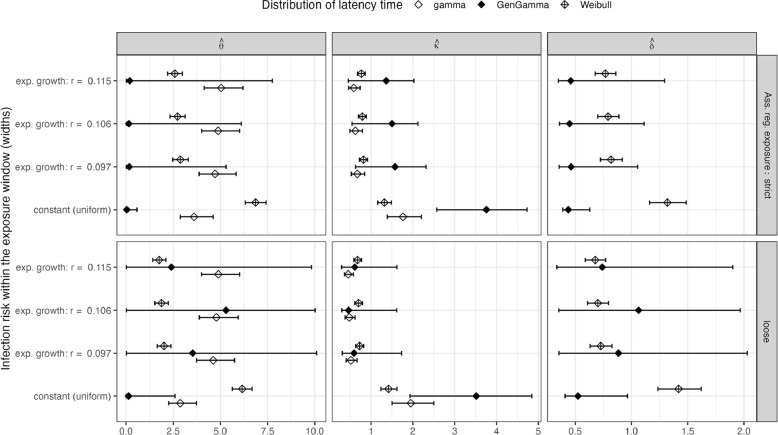
Parameter estimates with 95% credible intervals of the latency time distribution for SARS-CoV- 2. Parameters refer to the generalized gamma distribution with three parameters $\theta$, $\kappa$ and $\delta$ (columns) as proposed by Stacy and Mihram [19], of which the gamma ($\delta$ = 1) and Weibull ($\kappa$ = $\theta$) distribution are special cases. Estimates are given making two different assumptions for the risk of infection within the exposure window (within panel) and two different assumptions regarding the exposure window bounds (rows)

**Fig. 10:**
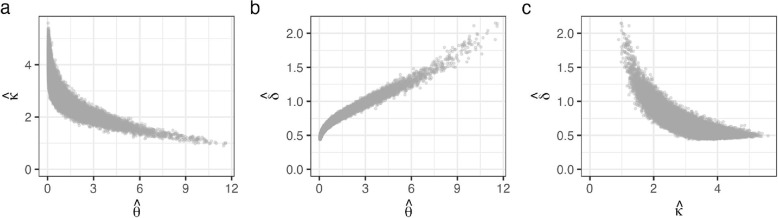
Bivariate posterior distribution of parameter pairs of the generalized gamma distribution for the latency time. Settings: strict assumption regarding exposure window and assuming a constant infection risk. Each dot represents one iteration (45,000 in total). Each subfigure (**a-c**) visualizes a different combination of parameters. Strong correlation between each parameter pair is present

The parameter estimates that characterize the distributions are reported in Appendix 5. The generalized gamma distribution may be overparameterized for the amount of information as present in the data (Figs. 9 and 10 in the Appendix 5). This may explain why the Weibull and gamma distribution give comparable results for the quantiles.

**Fig. 11:**
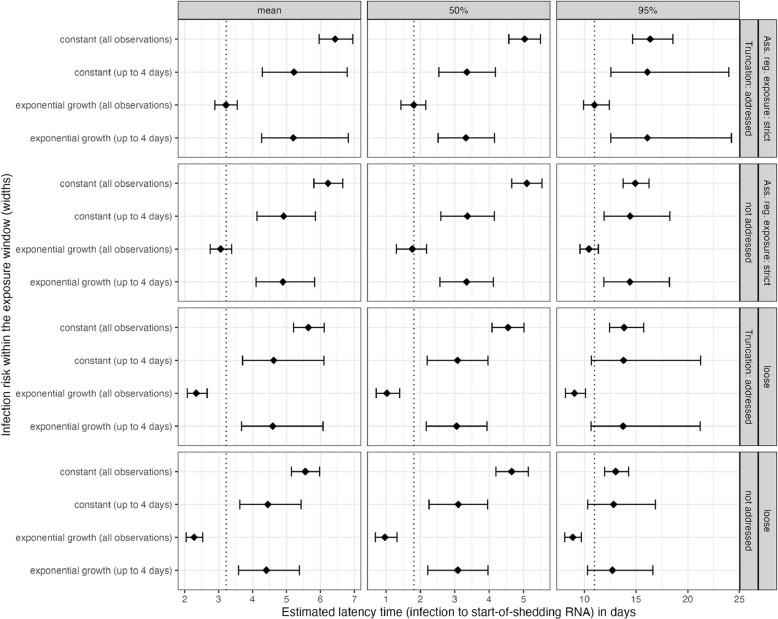
Estimated mean and percentiles for different assumptions regarding exposure and right truncation. Within each panel, the y-axis represents the assumed infection risk within the exposure window (r = 0.106 for exponential growth) and whether all observations or observations with exposure window $\le$ 4 days were selected for analysis. Rows correspond to the assumption regarding the exposure window bounds and whether truncation was addressed in the analysis. Left, middle and right column correspond to the estimated characteristic. All analyses assumed a generalized gamma distribution

The results of the sensitivity analyses are shown in Fig. 11 in the Appendix 6. Restricting the analysis to observations with a narrow exposure window ($\le$ 4 days; *N*= 176 using the strict choice and *N*= 134 using the loose choice) resulted in estimates that were larger than those obtained under the assumption of exponential growth and smaller than those assuming a constant infection risk. Not addressing truncation mostly impacted the higher percentiles. It led to slightly smaller estimates and a shrunken upper limit of the credible intervals for the 95 th percentile, especially if only the narrow exposure windows were used.

## Discussion

We estimated the SARS-CoV- 2 latency time distribution using a large and unique data set from Ho Chi Minh City, Vietnam. Covering the period from May to July 2021, almost all individuals were infected with the Delta variant. We can assume that they were mostly naive with respect to both infection and vaccination. They were mostly naive with respect to infection, because hardly any infections had occurred in Vietnam before May 2021. They were mostly naive with respect to vaccination, because only about 0.6% (*N*= 64,218) of the Ho Chi Minh City population of more than 10 million had received a vaccination dose by June 21, increasing to 923,050 by the end of June. In the data included for analysis, 65% (*N*= 1267) had the first positive test before June 21 and 98% (*N*= 1919) before July 1 st. Similarly in the country of Vietnam, the daily number of administered vaccination doses only started to increase more strongly from mid-June onwards, rising from 1.55 million doses to 4.06 million doses by July 12 th [26], on a total population size of almost 100 million.

Our estimates did not change much if the more strict Weibull or gamma distribution were chosen, or if we changed the exponential growth rate. Estimates were slightly smaller with the loose choice of the exposure window. The most likely explanation is that the loose choice mostly set the upper limit of the exposure window to a later calendar time, at least for the individuals for whom we had fairly accurate information with respect to both exposure and start-of-shedding. These are the individuals who have the largest impact on the value of the estimates; individuals with wide exposure and/or start-of-shedding window contribute little to nothing to the estimates.

Our estimates of the mean (3.22 days) and median (1.81 days) latency time are smaller than the earlier estimates for the Delta variant, while estimates for the 95 th percentile (10.98 days) are similar. However, estimates strongly depend on our assumptions regarding the risk of infection within the exposure window. We assumed an exponential increase in infection risk over calendar time. Since our data was collected during an outbreak, we considered this more realistic than a constant risk or an exponential decay as used in Xin et al. [6]. Yet, for some individuals, this exponential increase may not accurately represent their individual risk. The infection risk may be constant when only a single known individual is the source, as may be the case in household transmission [9]. When we restricted the analysis to individuals with a narrow exposure window width ($\le$4 days), the estimates became much larger with a mean of 5.20 (95% CrI 4.26; 6.82), a median of 3.32 (2.51; 4.15) and a 95 th-percentile of 16.10 (12.55; 24.23) days (Supplementary Table and Fig. 11 in the Appendix 6). However, restricting the analysis to the subset of the population with narrow exposure intervals may induce selection bias, for example due to differential recall [27, 28].

Our purpose was to estimate a population wide distribution. We did not have data on factors that explain variation in latency time, apart from sex and age. The Vietnamese population is relatively young, e.g. median age in our data set is 31 years (with 25 and 75 percentile of 23 and 43 years) and 32 countrywide. Our estimate of the latency time distribution may be similar to other low- and middle income countries with a similar age structure, but may differ from countries with a much older population.

Incubation time and latency time are closely related, except that 40.5% of the SARS-CoV- 2 infected individuals remain asymptomatic [29]. We could not estimate the incubation time distribution due to incomplete data on symptom onset. In a large study, the mean incubation time for the Delta variant was estimated to be 4.43 days (95% CI 4.36; 4.49) [30]. Our estimate of the latency time is shorter, which is to be expected given the possibility of pre-symptomatic spread of the infection [31].

Our analysis has several statistical strengths. We accounted for the doubly interval censored nature of our observations, as well as for the possibility of right truncation. We assumed an exponential increase in infection risk, which for our data is more realistic than a uniform distribution and we used a flexible generalized gamma distribution for the latency time distribution. Although estimates assuming a Weibull or gamma distribution and ignoring right truncation were similar in our data set, this may not be the case in other settings [9]. Only one other study on SARS-CoV- 2 latency time mentioned that truncation was addressed, but their likelihood formula differs from ours [6]. We provide the code of our estimation approach, and implemented it in an R package doublIn (on CRAN). Our code can also be used for incubation time estimation, as symptom onset is typically interval censored with a one-day window. Furthermore, it can be used to estimate other delay distributions with doubly interval censored data, such as the generation interval and serial interval.

The study also has some limitations. We already mentioned the sensitivity of the latency time estimates to the choice of the infection risk distribution within one’s exposure window. We did not have information to derive transmission trees and obtain a more accurate estimate of infection time. Second, we used start-of-shedding as the onset of infectiousness, but they may not align precisely. Third, as with all other studies on SARS-CoV- 2 latency and incubation time, data had not been collected for scientific purposes, but in order to control and contain spread of the virus. Hence, there was no pre-specified data collection procedure and we had no control over the completeness and correctness of the exposure window information. For example, unobserved transmission within quarantine facilities cannot be ruled out as some individuals quarantined with infected individuals in one room. When possible transmission within the quarantine facility was mentioned in the public health reports we took this into account in the choice of exposure window. But some of these possible transmissions may have gone unnoticed. A fruitful future direction would be to collect data in real time by using a digital questionnaire form that takes the scientific purpose into account, besides the typical purpose of controlling the outbreak. We developed such a questionnaire form, which is available via https://github.com/manhnguy/Contact-Tracing-for-Respiratory-Transmitted-Diseases. Answers to the questionnaire form can directly be imported into R, and we provide guidance on how to do so.

## Conclusion

We provide a new and flexible method to estimate of the latency time distribution for the SARS-CoV- 2 Delta variant. The code for our approach is openly available. To facilitate valid estimation in future outbreaks, further efforts to improve the quality of data collection and more research into correct model assumptions regarding infection are needed.

## Supplementary Information

Additional file 1. In a supplementary table (AdditionalFile1.pdf) we give the main estimates as numeric values. AdditionalFile2.csv contains the data to compute the growth rate. AdditionalFile3.csv contains the data to estimate the latency time distribution.

## Figures and Tables

**Fig. 7:**
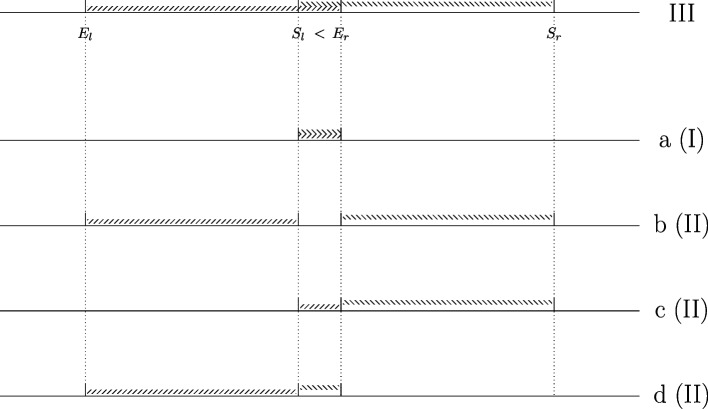
Illustration of data representation of partially overlapping exposure and start-of-shedding windows. To implement the frequentist approach, we decompose partially overlapping windows (type III in Fig. 2) into three distinct parts, one for the completely overlapping part (a) or three for the remaining non-overlapping parts (b, c and d). Part a is of type I in Fig. 2, whereas part b, c and d are of type II. Inspired by the source code in the coarseDataTools package, the sum of the likelihoods of part a, b, c and d together give the likelihood of an observation with partially overlapping windows

## Data Availability

Anonymised data supporting the conclusions of this article are included as additional files within the article. R/JAGS code and data to reproduce our analyses can be accessed via https://github.com/RonaldGeskus/LatencyTime.

## Appendix 1 Contact tracing and quarantine

Direct ($F_1$) and secondary ($F_2$) contacts of confirmed cases ($F_0$) were actively traced. Contact tracing typically concerned the period from 14 to 21 days before testing positive or symptom onset of the confirmed case, whichever event occurred first, until the time of quarantine or investigation. Contact tracing was bidirectional; it aimed not only to identify the infection chains (infectees) starting from the $F_0$ but also to trace back to the source that infected the $F_0$. Attendance at events known to have led to transmission was explicitly asked for. All $F_0$ individuals were hospitalized, while $F_1$ individuals and incoming passengers who tested negative were required to quarantine in a government-allocated facility. Secondary contacts ($F_2$) quarantined at home. The quarantine lasted for a minimum of 14 days since the last date of possible exposure. For $F_1$ contacts and incoming passengers the quarantine period was extended to three weeks between May 5 th to July 14 th, 2021 [32]. Individuals ended their quarantine if they still tested negative at the end of this period. If an $F_1$ individual tested positive, the person was transferred to the hospital, and all the contacts moved from $F_2$ to $F_1$ status.

## Appendix 2 Alternative analyses

We also explored a frequentist approach built upon R code by Ramjith et al. [16]. It was written for doubly interval censored observations with two non-overlapping or completely overlapping windows. We utilized the idea present in the source code of the R package coarseDataTools on representing the observation when exposure and start-of-shedding windows partially overlap and adapted the code by Ramjith *et al.* accordingly. Then, the contribution to the likelihood of two overlapping windows can be written as the sum of four distinct parts as shown in Fig. 7 in the Appendix 2. Each part has a likelihood as in the main text, i.e. Expression (1) for type (a), which now becomes

$$\begin{aligned} \int _{e = s_{il}}^{e_{ir}} \int _{s = e}^{e_{ir}} g(e|e_{il},e_{ir}) f(s-e)dsde. \end{aligned}$$

and Expression (2) for type (b),(c) and (d), which now become

$$\begin{aligned} \text {type b:} & \qquad \int _{e=e_{il}}^{s_{il}} \int _{s=e_{ir}}^{s_{ir}} g(e|e_{il},e_{ir}) f(s-e) dsde\\ \text {type c:} & \qquad \int _{e=s_{il}}^{e_{ir}} \int _{s=e_{ir}}^{s_{ir}} g(e|e_{il},e_{ir}) f(s-e) dsde\\ \text {type d:} & \qquad \int _{e=e_{il}}^{s_{il}} \int _{s_{il}}^{e_{ir}} g(e|e_{il},e_{ir}) f(s-e) dsde \end{aligned}$$

With our data set we faced convergence issues that we hypothesize to be related to local maxima in the likelihood.

In an attempt to move away from the assumption of a constant risk of infection, we considered exponential risk as described in the main text but also two variations. In the first approach the risk of infection over calendar time was estimated using the nonparametric maximum likelihood estimator for interval censored data (NPMLE; R package interval). In the second approach we estimated an infection time distribution within each of the three largest clusters separately. However, the NPMLE had a very limited amount of jumps, indicating that our data contained too much uncertainty regarding the moment of infection to choose one of these approaches.

## Appendix 3 Generalized gamma distribution

Stacy and Mihram [19] proposed the probability density function of the generalized gamma distribution as

4 $$\begin{aligned} f(t|\theta ,\kappa , \delta ) = \frac{ \frac{\delta }{\theta ^\kappa } }{\Gamma (\frac{\kappa }{\delta })}t^{\kappa - 1}e^{-(\frac{t}{\theta })^\delta } \end{aligned}$$

where $\theta$, $\kappa$, $\delta> 0$. The generalized gamma distribution contains the gamma distribution ($\delta = 1$), lognormal ($\frac{\kappa }{\delta } \rightarrow \infty$) and Weibull ($\kappa = \delta$) distribution as special cases. It has expected value

5 $$\begin{aligned} \theta \frac{\Gamma (\frac{\kappa + 1}{\delta } )}{\Gamma (\frac{\kappa }{\delta })} , \end{aligned}$$

and variance

6 $$\begin{aligned} \theta ^2 \left( \frac{\Gamma (\frac{\kappa + 2}{\delta })}{\Gamma (\frac{\kappa }{\delta })} - \left( \frac{\Gamma (\frac{\kappa +1}{\delta })}{\Gamma (\frac{\kappa }{\delta })}\right) ^2\right) . \end{aligned}$$

Table 1 shows how this parameterization relates to the software implementations of the generalized gamma distribution in the R package flexsurv [33] and in JAGS [24]. We also show the relation between the generalized gamma distribution and the gamma and Weibull distributions in the base R survival package.

**Table 1:** Three parameterizations of generalized gamma distribution. Gamma and Weibull as special cases in survival R package

Stacy	R flexsurv	JAGS, rjags	base R survival	
	gengamma.orig(shape,scale,k)	dgen.gamma( $r, \lambda , b$)	dgamma(shape, scale)	dweibull(shape, scale)
$\theta$	scale	$\frac{1}{\lambda }$	scale	scale
$\kappa$	shape $\cdot$ k	$r \cdot b$	shape	shape
$\delta$	shape	*b*	1	shape

For the frequentist approach discussed in Appendix 2, we used the relationship between the cumulative distribution functions of the gamma distribution $G(t|\theta , \kappa )$ with scale $\theta$ and shape $\kappa$ and the generalized gamma distribution $F(t|\theta , \kappa , \delta )$ as in Rubio [34], i.e.

7 $$\begin{aligned} F(t|\theta , \kappa , \delta ) = G\left(t^\delta |\theta ^\delta , \frac{\kappa }{\delta }\right), \end{aligned}$$

and the R functions provided by Rubio on his webpage.

## Appendix 4 Implementation of our model in JAGS

Our JAGS code is surprisingly concise, thanks to the implementation of both interval censoring and truncation in JAGS. Below we show the code for a model assuming an exponential increase of infection risk within the exposure window and a generalized gamma distribution for the latency time.

For every individual, both the time origin and the event time are interval censored. This means that each individual *i* has a start and stop time for both infection ($e_{il}$ and $e_{ir}$) and start-of-shedding ($s_{il}$ and $s_{ir}$). In the original data they are in calendar time. However, the assumption of both the uniform and the exponential distribution for the infection risk allows for subtracting the quantity $e_{il}$ from each value. Hence we have $\mathsf {ER[i]}=e_{ir}-e_{il}$, $\mathsf {SL[i]}=s_{il}-e_{il}$ and $\mathsf {SR[i]}=s_{ir}-e_{il}$, while the quantity $\mathsf {EL[i]}$ is not used.

The likelihood component as specified in Expressions (1) and (2) and in Appendix 2 contains a non-standard integral. However, computation of this integral can be circumvented. In the Markov Chain Monte Carlo procedure, the unobserved true infection time $\mathsf {E[i]}$ is repeatedly drawn from a pre-specified distribution. We can use the $\mathsf {T(0, ER[i])}$ construct to restrict its distribution to the individual’s exposure window. Note that for exponential growth we need to reverse the time scale; a uniform infection risk can be implemented without $\textsf {E\_star[i]}$, saving one line. The interval censored endpoint is implemented via the function dinterval, with limits determined by $\mathsf {SL[i]-E[i]}$ and $\mathsf {SR[i]-E[i]}$. Since we have two observation times, $\mathsf {type\_S[i]}$ is allowed to take the values 0 (for left censored data), 1 (for truely interval censored data) and 2 (for right censored data). We made every individual to have an interval of start-of-shedding, hence $\mathsf {type\_S[i]=1}$ for every individual. The right truncation at $T_{i,\textrm{end}}$ is represented via truncation at $\mathsf {Trunc[i]-E[i]}$.

The parameterization of the generalized gamma distribution in JAGS differs from the one proposed by Stacy and Mirham [19] (see Table 1). For the parameters of the generalized gamma distribution we use noninformative normal priors, truncated to positive half line. For the exponential growth factor r, a normal distribution, with parameters given by the external estimate (r_est) and its standard error (r_se) as input data, is assumed. Note that JAGS works with precision rather than variance.

We verified the validity of the implementation with right truncated data by means of a simulation study. Data were generated assuming a random exposure window width varying from one to five days and a constant risk of infection within the window. We chose a gamma distribution for the latency time as estimated by Xin et al. [6] (shape = 4.05, rate = 0.74; median 5.03; 95 th percentile 10.57). The endpoint was observed up to the day accurate. Quarantine started at the end of the exposure window. Individuals were tested at the end of quarantine. We simulated three scenarios with respect to truncation due to end of quarantine: i) individuals left quarantine 5, 10, 15, 20 or 25 days after entering, each occurring with equal probability; ii) quarantine lasted 7 days for all individuals; iii) quarantine lasted 7 or 14 days (with equal probability). We generated 500 data sets per scenario and 1000 infected individuals per data set before truncation. Only those individuals who tested positive before exiting quarantine were included in the analyses; the others remained unobserved. Our simulation code can be accessed via the example section the R help file for function Estimate_doublIn in our R package doublIn.

Figure 8 in the Appendix 4 shows that our JAGS code adequately addresses truncation. Note that the difference between the analyses with and without correction for truncation is small when the level of truncation is very mild (14 or 21 days) whereas the difference is large when the level of truncation varies (5, 10, 15, 20, or 25 days) or is consistently strong (7 days). For quarantine scenario ii), 150 of the 500 data sets did not provide a model fit for at least one of the two models (addressing truncation or not), probably due to too little information present in the data set.

## Appendix 5 Parameter estimates of latency time distribution

Although the uncertainty in the mean and percentiles based on the generalized gamma distribution is comparable to the gamma and Weibull distributions, the uncertainty in the parameter estimates is much larger for the generalized gamma distribution (Fig. 9 in the Appendix 5). Apparently, the coarse nature of the data does not allow to clearly distinguish between the different parameters, which can also been seen by the strong collinearity between the parameters in the posterior distribution (Fig. 10 in the Appendix 5).

## Appendix 6 Sensitivity analyses

Figure 11 in the Appendix 6 presents the latency time estimates from sensitivity analyses regarding the observations that were included and whether truncation was addressed. When only observations with narrow exposure windows of 4 days at most were used, estimates are in-between the estimates based on the two assumptions regarding the infection risk when all observations were included for analysis, but with much wider credible intervals due to smaller sample size (*N*= 176 using the strict choice and *N*= 134 using the loose choice). The difference between assuming exponential growth and assuming a constant risk of infection disappears.
