# Supplementary material for: The latency time of SARS-CoV- 2 Delta variant in infection- and vaccine-naive individuals from Vietnam
Source: BMC Infect Dis. 2025 Apr 12;25:515. doi: 10.1186/s12879-025-10898-3 (PMC11993988; doi:10.1186/s12879-025-10898-3)
Supplement: Supplementary file 1 — Additional file 1. In a supplementary table (AdditionalFile1.pdf) we give the main estimates as numeric values. AdditionalFile2.csv contains the data to compute the growth rate. AdditionalFile3.csv contains the data to estimate the latency time distribution. [file 12879_2025_10898_MOESM1_ESM.zip › AdditionalFile1.pdf]

Supplementary Table: Estimates under different assumptions regarding the analysis. All models reported assume that the latency time distribution follows a generalized gamma distribution. *Abbreviations:* Inf. Risk: assumption with respect to infection risk in exposure window. Exp. window: (assumption regarding) exposure window. Narrow exp.w.: observations with an exposure window width $\leq$ 4 days. CrI: credible interval.

Truncation	Exp. window	Inf. Risk	Data	Mean	(95% CrI)	50%	(95% CrI)	95%	(95% CrI)	$\hat{\theta}$	(95% CrI)	$\hat{\kappa}$	(95% CrI)	$\hat{\delta}$	(95% CrI)
not addressed	loose	constant (uniform)	all	5.56	(5.15; 5.98)	4.65	(4.19; 5.13)	13.03	(11.96; 14.28)	0.84	(0.07; 7.24)	2.83	(1.33; 4.45)	0.73	(0.5; 1.66)
			narrow exp.w.	4.45	(3.63; 5.43)	3.10	(2.25; 3.95)	12.83	(10.33; 16.88)	0.04	(0.01; 10.17)	2.60	(0.67; 3.93)	0.42	(0.35; 2.00)
		exp. growth: r = 0.097	all	2.36	(2.12; 2.62)	1.05	(0.76; 1.42)	9.02	(8.23; 9.85)	7.74	(2.76; 10.8)	0.39	(0.29; 0.70)	1.49	(0.84; 2.35)
			narrow exp.w.	4.41	(3.59; 5.38)	3.09	(2.21; 3.97)	12.71	(10.31; 16.65)	0.09	(0.02; 12.83)	2.28	(0.52; 3.68)	0.46	(0.37; 3.06)
		exp. growth: r = 0.106	all	2.28	(2.05; 2.53)	0.97	(0.69; 1.32)	8.87	(8.09; 9.69)	7.66	(2.18; 10.63)	0.38	(0.29; 0.72)	1.46	(0.77; 2.26)
			narrow exp.w.	4.41	(3.59; 5.38)	3.09	(2.22; 3.97)	12.71	(10.31; 16.63)	0.12	(0.02; 12.71)	2.23	(0.53; 3.62)	0.47	(0.37; 3.00)
		exp. growth: r = 0.115	all	2.20	(1.98; 2.45)	0.88	(0.64; 1.20)	8.75	(7.97; 9.56)	7.74	(2.87; 10.53)	0.36	(0.28; 0.62)	1.46	(0.83; 2.21)
			narrow exp.w.	4.39	(3.56; 5.35)	3.09	(2.18; 3.99)	12.64	(10.31; 16.44)	0.31	(0.02; 13.64)	1.96	(0.48; 3.51)	0.54	(0.37; 3.54)
	strict	constant (uniform)	all	6.22	(5.81; 6.66)	5.09	(4.65; 5.54)	14.93	(13.76; 16.27)	0.15	(0.02; 1.24)	3.72	(2.49; 4.84)	0.53	(0.43; 0.77)
			narrow exp.w.	4.92	(4.13; 5.85)	3.37	(2.59; 4.15)	14.43	(11.91; 18.27)	0.05	(0.02; 1.50)	2.49	(1.39; 3.55)	0.42	(0.36; 0.71)
		exp. growth: r = 0.097	all	3.21	(2.88; 3.55)	1.98	(1.48; 2.38)	10.49	(9.63; 11.43)	1.60	(0.02; 9.27)	1.08	(0.44; 2.51)	0.72	(0.38; 1.62)
			narrow exp.w.	4.89	(4.11; 5.81)	3.34	(2.57; 4.12)	14.41	(11.91; 18.17)	0.07	(0.02; 2.51)	2.38	(1.21; 3.39)	0.44	(0.37; 0.81)
		exp. growth: r = 0.106	all	3.06	(2.75; 3.39)	1.76	(1.30; 2.18)	10.43	(9.57; 11.35)	4.05	(0.09; 9.96)	0.72	(0.40; 1.92)	0.96	(0.44; 1.72)
			narrow exp.w.	4.89	(4.1; 5.83)	3.34	(2.57; 4.12)	14.41	(11.89; 18.24)	0.05	(0.01; 2.17)	2.47	(1.26; 3.54)	0.42	(0.36; 0.77)
		exp. growth: r = 0.115	all	2.93	(2.64; 3.24)	1.58	(1.15; 2.01)	10.37	(9.52; 11.28)	5.76	(0.17; 10.52)	0.57	(0.36; 1.61)	1.12	(0.47; 1.82)
			narrow exp.w.	4.89	(4.1; 5.81)	3.34	(2.56; 4.11)	14.41	(11.9; 18.20)	0.07	(0.02; 2.27)	2.38	(1.24; 3.41)	0.44	(0.36; 0.78)
addressed	loose	constant (uniform)	all	5.64	(5.21; 6.11)	4.55	(4.08; 5.01)	13.84	(12.43; 15.73)	0.13	(0.02; 2.59)	3.51	(1.93; 4.84)	0.52	(0.41; 0.96)
			narrow exp.w.	4.62	(3.71; 6.10)	3.08	(2.20; 3.96)	13.79	(10.67; 21.24)	0.05	(0.02; 7.26)	2.31	(0.83; 3.58)	0.42	(0.34; 1.40)
		exp. growth: r = 0.097	all	2.44	(2.16; 2.77)	1.16	(0.80; 1.52)	9.15	(8.25; 10.25)	3.53	(0.02; 10.09)	0.59	(0.31; 1.73)	0.88	(0.35; 2.03)
			narrow exp.w.	4.59	(3.67; 6.06)	3.06	(2.18; 3.95)	13.7	(10.61; 21.03)	0.06	(0.02; 10.64)	2.20	(0.64; 3.53)	0.43	(0.34; 2.13)
		exp. growth: r = 0.106	all	2.34	(2.08; 2.66)	1.03	(0.72; 1.40)	9.03	(8.16; 10.10)	5.30	(0.03; 10.02)	0.46	(0.30; 1.61)	1.06	(0.35; 1.97)
			narrow exp.w.	4.60	(3.68; 6.08)	3.05	(2.17; 3.94)	13.75	(10.64; 21.20)	0.05	(0.02; 8.76)	2.24	(0.73; 3.53)	0.42	(0.34; 1.65)
		exp. growth: r = 0.115	all	2.30	(2.03; 2.63)	0.98	(0.67; 1.32)	8.94	(8.03; 10.20)	2.40	(0.02; 9.83)	0.61	(0.30; 1.62)	0.74	(0.34; 1.90)
			narrow exp.w.	4.59	(3.67; 6.04)	3.06	(2.18; 3.94)	13.68	(10.61; 20.98)	0.06	(0.02; 10.41)	2.19	(0.65; 3.51)	0.43	(0.34; 2.04)
	strict	constant (uniform)	all	6.43	(5.96; 6.95)	5.03	(4.57; 5.49)	16.37	(14.68; 18.57)	0.04	(0.01; 0.58)	3.76	(2.57; 4.73)	0.44	(0.39; 0.63)
			narrow exp.w.	5.22	(4.28; 6.79)	3.35	(2.54; 4.18)	16.10	(12.56; 23.98)	0.05	(0.01; 1.17)	2.21	(1.28; 3.26)	0.4	(0.33; 0.64)
		exp. growth: r = 0.097	all	3.32	(3.00; 3.65)	1.94	(1.57; 2.30)	11.07	(10.02; 12.45)	0.17	(0.02; 5.30)	1.57	(0.63; 2.31)	0.46	(0.36; 1.05)
			narrow exp.w.	5.20	(4.26; 6.79)	3.32	(2.51; 4.16)	16.11	(12.56; 24.05)	0.05	(0.01; 1.22)	2.20	(1.25; 3.25)	0.4	(0.33; 0.65)
		exp. growth: r = 0.106	all	3.22	(2.89; 3.55)	1.81	(1.44; 2.16)	10.98	(9.91; 12.41)	0.15	(0.02; 6.11)	1.50	(0.54; 2.12)	0.45	(0.36; 1.11)
			narrow exp.w.	5.20	(4.26; 6.82)	3.32	(2.51; 4.15)	16.10	(12.55; 24.23)	0.05	(0.02; 1.02)	2.21	(1.30; 3.24)	0.4	(0.33; 0.62)
		exp. growth: r = 0.115	all	3.11	(2.78; 3.45)	1.67	(1.30; 2.03)	10.88	(9.81; 12.35)	0.20	(0.02; 7.75)	1.36	(0.46; 2.02)	0.46	(0.35; 1.29)
			narrow exp.w.	5.20	(4.26; 6.80)	3.32	(2.51; 4.15)	16.09	(12.54; 24.12)	0.05	(0.01; 1.35)	2.22	(1.24; 3.27)	0.4	(0.33; 0.66)
